# Mapping the mind of a fly

**DOI:** 10.7554/eLife.62451

**Published:** 2020-10-08

**Authors:** Jason Pipkin

**Affiliations:** Department of Biology, Brandeis UniversityWalthamUnited States

**Keywords:** connectome, brain regions, cell types, graph properties, connectome reconstuction methods, synapse detection, *D. melanogaster*

## Abstract

Scientists have created the most detailed map of the fruit fly brain to date, identifying over 25,000 neurons and 20 million synapses.

**Related research article** Scheffer LK, Xu CS, Januszewski M, Lu Z, Takemura SY, Hayworth KJ, Huang GB, Shinomiya K, Maitlin-Shepard J, Berg S, Clements J, Hubbard PM, Katz WT, Umayam L, Zhao T, Ackerman D, Blakely T, Bogovic J, Dolafi T, Kainmueller D, Kawase T, Khairy KA, Leavitt L, Li PH, Lindsey L, Neubarth N, Olbris DJ, Otsuna H, Trautman ET, Ito M, Bates AS, Goldammer J, Wolff T, Svirskas R, Schlegel P, Neace E, Knecht CJ, Alvarado CX, Bailey DA, Ballinger S, Borycz JA, Canino BS, Cheatham N, Cook M, Dreher M, Duclos O, Eubanks B, Fairbanks K, Finley S, Forknall N, Francis A, Hopkins GP, Joyce EM, Kim S, Kirk NA, Kovalyak J, Lauchie S, Lohff A, Maldonado C, Manley EA, McLin S, Mooney C, Ndama M, Ogundeyi O, Okeoma N, Ordish C, Padilla N, Patrick CM, Paterson T, Phillips EE, Phillips EM, Rampally N, Ribeiro C, Robertson MK, Rymer JT, Ryan SM, Sammons M, Scott AK, Scott AL, Shinomiya A, Smith C, Smith K, Smith NL, Sobeski MA, Suleiman A, Swift J, Takemura S, Talebi I, Tarnogorska D, Tenshaw E, Tokhi T, Walsh JJ, Yang T, Horne JA, Li F, Parekh R, Rivlin PK, Jayaraman V, Costa M, Jefferis GS, Ito K, Saalfeld S, George R, Meinertzhagen I, Rubin GM, Hess HF, Jain V, Plaza SM. 2020. A connectome and analysis of the adult *Drosophila* central brain. *eLife* **9**:e57443. doi: 10.7554/eLife.57443

Every thought, feeling and action emerges from the electrical interplay of billions of neurons in the brain – wired together by an intricate network of cables that connect through hundreds of billions of synapses. Therefore, to fully understand how the brain works we need to consider all parts of the brain and the connections between them.

A connectome is a comprehensive map of the structural and functional neural connections in the brain that enables scientists to explore and compare different pathways, circuits and regions. Creating such a map is a difficult endeavor: neurons are minuscule, and their extensive branches are even smaller (for example, neuronal branches in the brain of a fruit fly are often thinner than 50 nm – about a thousandth of the width of a human hair).

To achieve such a high resolution, ultrathin layers of brain tissue are imaged with an electron microscope, and the neurons and their connections are reconstructed in 3D. This is no small undertaking, and in the case of the roundworm *Caenorhabditis elegans* (whose brain only consists of 302 neurons), it took the better part of a decade to generate a comprehensive connectome (White et al., 1986). This dissuaded attempts to create connectomes for larger brains, until the technological advances in microscopy and computer vision finally caught up with demand (Denk and Horstmann, 2004; Heymann et al., 2006; Januszewski et al., 2018).

Nowadays, multiple efforts have been underway to conquer the next model organism, the fruit fly *Drosophila melanogaster*. In a space smaller than a pinhead, the brain of a fruit fly contains over 100,000 neurons with around 100 million synapses (Simpson, 2009). So far, 3D reconstructions of its brain have tended to either be sparse or limited to small sub-regions (Eichler et al., 2017; Takemura et al., 2017; Zheng et al., 2018). Now, in eLife, Louis Scheffer, Stephen Plaza (both Janelia Research Campus, HHMI) and colleagues – including Scheffer, Shan Xu, Michal Januszewski, Zhiyuan Lu, Shin-ya Takemura, Kenneth Hayworth, Gary B. Huang and Kazunori Shinomiya as joint first authors – report that they have created the largest high-resolution connectome in any animal to date (Scheffer et al., 2020).

The researchers (who are based in the US, Canada, Switzerland, Japan, Germany and UK) generated a connectome for one half of the largely symmetrical fly brain, comprising over 25,000 neurons and 20 million synapses (Figure 1A). To achieve this, Scheffer et al. analyzed 3D reconstructions of the neurons and their synapses using machine learning algorithms and more than 50 person-years of work to proofread the computer-generated map. This involved transforming more than 20 terabytes of raw image data into a 26-megabyte network diagram.

**Figure 1.**
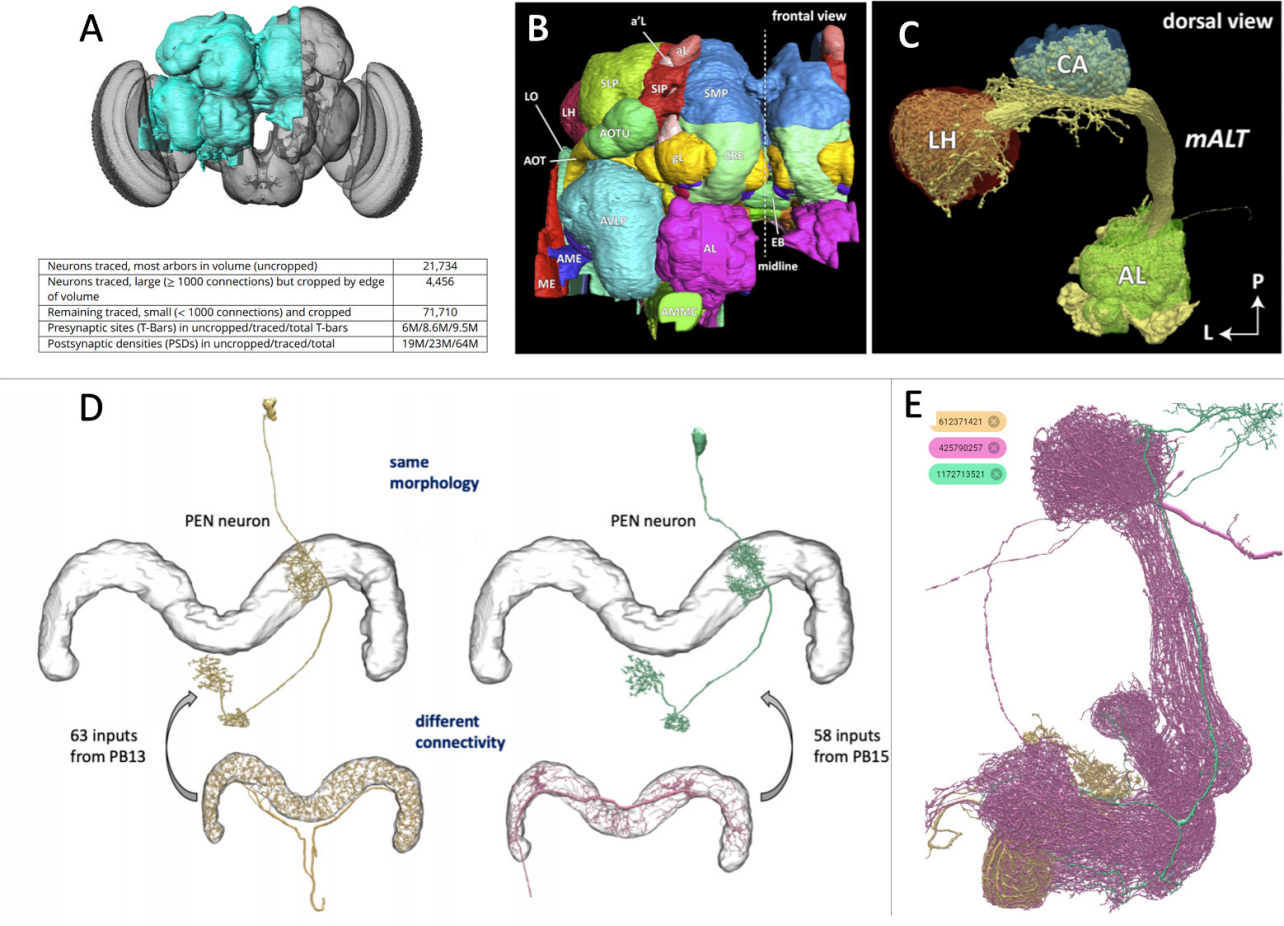
The connectome of a fruit fly. (**A**) Scheffer et al. have used machine learning to reconstruct half of the fruit fly brain (area shown in teal), providing the most comprehensive connectome of any animal to date. (**B**) The different regions in the brain were labelled according to known brain maps. The fly brain can be subdivided based on tracts of neuronal branches and large tangles called neuropils. Any given neuron might innervate several neuropils, and neurons are often identified by the neuropils they make synapses with. (**C**) Neuropils of the odor processing center (the antennal lobe AL, the calyx CA and the lateral horn LH) and the neurons (yellow) that connect them. (**D**) The connectome can differentiate neurons into separate types that would have been difficult to distinguish using light microscopy due to their similar anatomy. (**E**) The connectome contains a complete list of all neurons and their synapses, making it possible to search for specific neurons and their connections. For example, the database shows that a neuron from a region called the mushroom body (shown in tan) connects to two other neurons in this neuropil (shown in magenta and teal).

Image credit: Figure 1A–D adapted from Scheffer et al., 2020 (CC BY 4.0), Figure 1E obtained from the online portal Neuprint (CC0).

A good map should be informative, accurate and usable. Towards that end, Scheffer et al. have labelled regions according to existing atlases of the fly brain (Figure 1B). In addition, they subdivided the brain based on the tracts of neuronal branches and large tangles called neuropils, which are thought to be sites of local computation (Figure 1C). With regions delineated in this way, individual neurons could be categorized based on the neuropils they connect to. This is particularly useful for distinguishing neurons that have similar anatomies, which would be difficult to tell apart using light microscopy alone (Figure 1D). Moreover, the connectome is freely available at the online portal ‘Neuprint’, and anyone can search the database for a neuron of interest, observe the images it was traced from, plot it in 3D and see which brain regions it interacts with (Figure 1E).

Scheffer et al. were also able to quantify how highly interconnected the fly brain is. For example, the path distance between neurons (i.e., how many neurons there are between a connected pair) is low – three quarters of neurons were linked by three or fewer interneurons. Moreover, they demonstrated that the different neuropils are indeed segregated electrically, suggesting that the same neuron can perform separate computations in different regions at the same time.

Of course, no map is perfect. Most branches traced belong to yet unidentified neurons (presumably the ones that reside outside the borders of the sequenced brain). Many of the tiniest twigs that are difficult for both human and machine eyes to trace may have been missed or lie ‘disconnected’ from their true parent neuron, which may reside in the other unmapped half of the brain. This could potentially reduce the number of synapses between two cells.

Scheffer et al. have pledged to improve and update their connectome over time – after all, another half of the fly brain remains to be analyzed. Nevertheless, the current map marks a significant increase in scale over the one of *C. elegans* and will undoubtedly help unravel the neurological basis underlying a fly’s behavior. And it may bring us one step closer to creating connectomes of larger animals, including vertebrates.
